# The Early Childhood Obesity Prevention Program (ECHO): an ecologically-based intervention delivered by home visitors for newborns and their mothers

**DOI:** 10.1186/s12889-015-1897-9

**Published:** 2015-06-24

**Authors:** Michelle M. Cloutier, James Wiley, Zhu Wang, Autherene Grant, Amy A. Gorin

**Affiliations:** Department of Pediatrics, University of Connecticut Health Center, Farmington, CT USA; Children’s Center for Community Research, Connecticut Children’s Medical Center, 282 Washington St, Hartford, CT 06106 USA; Department of Research, Connecticut Children’s Medical Center, 282 Washington St, Hartford, CT 06106 USA; Department of Psychology, Center for Health, Intervention and Prevention, University of Connecticut, 2006 Hillside Road, Unit 1248, Storrs, CT 06269-1248 USA

**Keywords:** Latino, African American, Low-income, Stimulus control, Primary prevention, Family wellness, Newborns

## Abstract

**Background:**

Obesity is a major problem in the United States, particularly among socio-economically disadvantaged Latino and Black children. Effective interventions that can be disseminated to large numbers of at-risk children and their families are needed. The goals of the Early Childhood Obesity Prevention Program (ECHO) are to examine the 12-month efficacy of a primary obesity prevention program targeting the first year of life that is delivered by home visitors and that engages mothers as agents of change to modify their own behavior and their infant’s behavior through education and skill-building around nutrition, physical activity, and wellness, and then “echoes” her training with linkages to neighborhood programs and resources.

**Methods/Design:**

Six family centers located in low-income neighborhoods in Hartford, CT were randomized into control and intervention neighborhoods. Fifty-seven mothers were recruited either prenatally or shortly after delivery into the Nurturing Families Network home visitation program; 27 lived in a control neighborhood and received the standard home visitation program and 30 lived in an intervention neighborhood and received both the standard home visitation program and the ECHO intervention. The intervention increases maternal skills in goal-setting, stimulus control and problem-solving, engages family members to support changes, links mothers to neighborhood resources and is embedded in the standard home visitation program. ECHO targets include breastfeeding, solids, juice and sugar-sweetened beverages, routines for sleep and responding to infant cues, television/screen time, and maternal diet and physical activity. We hypothesize that infants in ECHO will have been breastfed longer and exclusively, will have delayed introduction of solids and juice, have longer sleep duration, decreased television/screen time and a lower weight for length *z*-score at 12 months, and their mothers will have greater fruit and vegetable consumption and higher levels of physical activity.

**Discussion:**

ECHO will provide important information about whether an enhanced behavior change curriculum integrated into an existing home visitation program, focused on the mother as the agent of change and linked to neighborhood resources is effective in changing energy balance behaviors in the infant and in the mother. If effective, the intervention could be widely disseminated to prevent obesity in young children.

**Trial Registration:**

ClinicalTrials.gov NCT02052518 January 30, 2014.

## Background

The prevalence of obesity in the United States has tripled in the past 40 years [1] with disproportionately higher rates in low-income children of color [2–4]. Socioeconomic and racial/ethnic disparities in rates of obesity appear during the preschool years [3]. Not surprisingly, children with an elevated body mass index before 5 years of age are at increased risk of becoming obese adults, suggesting a need to intervene early in life [5].

Many childhood obesity prevention efforts have focused on school-age children [6]. There is a small but growing body of literature on obesity prevention in young children [7–9]. These studies in young children suggest that parental involvement and engaging parents as agents of change are keys to early obesity prevention [10, 11]. One recent pilot study educated mothers to recognize infant cues and to delay the introduction of solids [11]. A second study used home visitors and incorporated maternal education into the *Parents as Teachers* program to increase fruit and vegetable consumption; they demonstrated a reduced weight for length and changes in parental behavior [12].

**Fig. 1:**
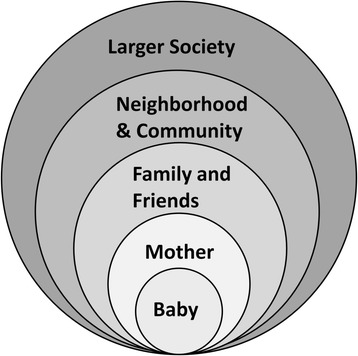
Ecological model of obesity prevention program

Thus, parental education and maternal behavior change appear to be important components of obesity prevention. They may not, however, be sufficient to prevent obesity if mothers do not have access to, cannot afford, or don’t know how to prepare quality food and/or don’t feel safe in their community. Ecological models of obesity (Fig. 1) recognize the broader contextual influences on individuals including families, neighborhoods, culture and the greater society. Interventions using an ecological model support families to sustain change and, in adults, have been shown to promote greater weight loss [13].

Home visitors can support behavior change effectiveness and can reach high-risk pregnant women and mothers during this early and critical period in a culturally responsive way [14–16]. National home visitation programs provide health and psychosocial-related services to at-risk pregnant women and mothers of infants and toddlers [17, 18]. Home visitors have been used to improve self-management of diabetes and asthma and a few trials of home visitation for the prevention of obesity are currently on-going [19–22].

The Nurturing Families Network (NFN) is a nationally recognized, evidence-based home visitation program with credentialed educators, and in Connecticut, is a program of the Children’s Trust Fund. The NFN Home Visitation Program uses two evidence-based curricula (*Parents as Teachers-Born to Learn* (PAT) and *Partners for a Healthy Baby* (Partners)) and a culturally diverse workforce [23, 24]. Both curricula achieve positive parenting and child outcomes, prevent child abuse, and enhance child development [25, 26]. The curricula provide some education on breastfeeding (2–3 h), introduction of solids, and infant routines, but the content is primarily education-focused and mothers are not taught behavioral skills to implement recommended behaviors. The effect of the NFN Home Visitation Program on maternal obesogenic behaviors and its role in obesity prevention in young children are not known.

Obesity prevention programs that begin before habits have become established and that use home visitors and a developmental perspective to address environmental and community contextual issues in a culturally responsive way may be successful in preventing obesity in young children. These interventions, however, need to be delivered early in life since rapid weight gain in the first 6 months of life predicts future obesity in children, independent of birth weight [27–31].

Potential goals for interventions in children in the first year of life include increasing breastfeeding, reducing sugar-sweetened beverage (SSB)/juice consumption, delaying the introduction of solids, limiting television/screen time, establishing infant routines around sleep, and recognizing infant cues to hunger and satiety. Breastfeeding decreases initial weight gain and is one strategy for reducing childhood overweight [32], although recent studies [33–37] have not found an obeso-protective effect. Breast milk, however, is the best nourishment for infants, and is a low-cost, readily available nutrient [38]. The American Academy of Pediatrics recommends no solids and no juice in the first 6 months of life but, in the United States, by 6 months of age, 50 % of infants consume sugar-sweetened beverages/juice daily [39], and 65 % consume solids, usually cereals [40]. Early SSB consumption has been associated with obesity [41] while delaying the introduction of solids to 6 months of age and not mixing cereal in bottles reduces mean weight for length [42]. Television viewing is obesogenic [43] and the American Academy of Pediatrics recommends no television/screen time for children less than 2 years and no television in the bedroom [44]. Helping parents to establish sleep routines and to recognize cues for hunger and satiety are obeso-protective while infant sleeping duration less than 11 h per day is associated with rapid weight gain by 6 months [45, 46].

Understanding the context in which obesogenic behaviors occur, helping parents of very young children to establish positive behaviors, and linking families to community resources could prevent the development of obesity in young children. These goals are the foundation of an intervention called the *Early Childhood Obesity Prevention Program (ECHO): Building Healthier Families and Communities.* ECHO is a randomized, controlled trial designed to examine the efficacy at 12 months of a home visitation program that engages mothers as agents of change to modify their own behavior and their infant’s behavior through education and skill building in the areas of nutrition, physical activity, and wellness, and then “echoes” her training with linkages to neighborhood programs (e.g., coupons to purchase fruits and vegetables at grocery stores and farmer’s markets, parenting classes at Family Centers, neighborhood cooking classes). ECHO recruits pregnant women and new mothers who have agreed to participate in NFN’s home visitation program and provides them with an enhanced curriculum that increases maternal skills in goal-setting, problem-solving, and stimulus control to target four specific infant-related behaviors (breastfeeding, SSB/juice consumption, introduction of solids, television/screen time and infant routines and cues around sleep and hunger and satiety) and follows the mother-infant dyad until the infant is 12 months of age. The program also provides education and linkages to community resources to improve the mother’s health, nutrition and physical activity. The study’s focus on newborns and mothers as agents of change adds to the small literature on obesity prevention in this critical developmental window.

## Methods/Design

### Overview and hypotheses

Pregnant women or new mothers within one month of delivery who reside in one of six urban neighborhoods in Hartford, CT and were recruited into the NFN program were invited to participate in ECHO. These six neighborhoods were chosen because they are served by a Brighter Future Family (BFF) Center that is linked to the Nurturing Families Network home visitation program. Brighter Future Family Centers provide early childhood education programs and parenting classes designed to improve parents’ knowledge of child development and nurturing skills, support in earning a high school degree, understanding and speaking English, computer use and financial literacy and family play groups. Centers also provide information to parents about other community, city, state and national resources that might help them.

The six Brighter Future Family (BFF) Centers in Hartford, Connecticut and their NFN programs were paired by neighborhood socioeconomic and racial/ethnic characteristics (Table 1) and were randomly assigned to receive either the standard NFN home visitation program (n = 3) or the enhanced NFN home visitation program (NFN+, n = 3). The goal was to recruit 60 mothers and their newborns (10 from each BFF Center and its corresponding neighborhood) for a total of 30 mother/newborn dyads from an intervention BFF Center and its neighborhood and 30 dyads from a control BFF Center and its neighborhood. All dyads received the standard NFN curriculum using PAT and Partners curricula with weekly home visits. In addition, the NFN+ dyads received an enhanced curriculum in the areas of goal-setting, problem-solving, stimulus control and skill-building related to nutrition, physical activity, cues, and routines as well as enhanced neighborhood community services including coupons for Farmer’s Markets and grocery stores, cooking classes, and exercise classes.

**Table 1:** Demographics of neighborhood pairs selected for randomization^a^

Neighborhood pair	1	2	3
Average family income	$28,191	$46,730	$33,697
Poverty rate^b^	42 %	14 %	34 %
Number of births (2010)	466	222	311
Total population (2010)	19,655	20,308	15,512
% Hispanic	50 %	32 %	41 %
% Black	32 %	45 %	44 %

^a^ http://www.hartfordinfo.org/Snapshots/neighborhood. Last accessed May 10, 2015

^b^City of Hartford Department of Health and Human Services. *A Community Health Needs Assessment.* Hartford, CT: City of Hartford Department of Health and Human Services;2012

We hypothesized that at 6 and 12 months, as compared to NFN infants, NFN+ infants would have been breastfed longer and more often exclusively, would drink less sugar-sweetened beverages/juice, would have begun to ingest solids at an older age, and would sleep more hours/day, have more established sleep/wake routines, greater soothability and have fewer hours of television viewing/day. Secondary hypotheses of the study included that at 6 and 12 months, as compared to NFN families, NFN+ families would purchase, cook and serve more fruits and vegetables, demonstrate greater utilization of BFF Centers and community resources, and NFN+ infants would be less likely to have a weight for length greater than the 85^th^ percentile. The study protocol was approved by the Institutional Review Board at Connecticut Children’s Medical Center, was sponsored by the Hartford Childhood Wellness Alliance, and was funded by the National Institutes of Health (NICHD R21 HD073966-A01).

### Participants

Women who were either prenatal or had just delivered a baby were screened for eligibility for the NFN program using the Revised Early Identification Screening Tool (Reid). The Reid screen consists of 17 items including poverty, unemployment, single head of household, substance abuse, psychiatric care or late prenatal care with a positive Reid screen score being 3 or more affirmative responses. Final eligibility for the NFN Home Visitation Program was determined using results from the Kempe Family Scale which was administered during the first home visit [47, 48] by the NFN Supervisor. Prenatal women enrolled in NFN were further screened for eligibility in ECHO; final determination of eligibility occurred after the birth of the infant. Inclusion criteria for ECHO included singleton birth greater than 34 weeks gestation, any maternal race or ethnicity, no chronic conditions that could affect the growth or development of the infant and residence at the time of the infant’s birth in an ECHO-designated neighborhood. Infants with major malformations, admission to the neonatal intensive care unit or a prolonged hospital stay or infants who were small for gestational age and required special or supplemental nutrition were excluded.

**Fig. 2:**
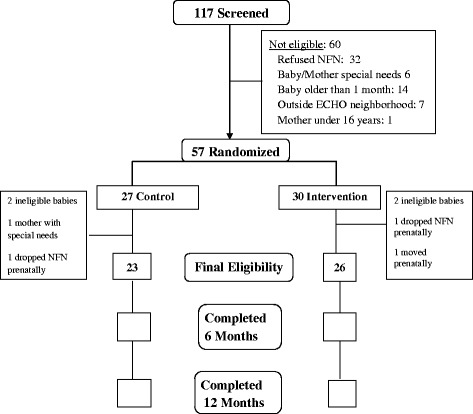
Study recruitment

Between November 2013 and December 2014, 117 mothers were screened for enrollment in ECHO and 57 met all initial eligibility criteria, provided consent, and were randomized into either the intervention (n = 30) or control (n = 27) arms (Fig. 2). After the birth of their newborn, 49 dyads continued to meet all final eligibility criteria. Of the 8 dyads that dropped out after providing consent, 2 left the NFN program prenatally, 4 had infants who did not meet eligibility criteria, 1 mother moved out of the area prenatally and 1 mother had special needs that precluded her participation in the study. The demographics of the 49 maternal-infant dyads that met all eligibility criteria are shown in Table 2.

**Table 2:** Demographics of mother infant dyads

	Control (n = 23) (%)	Intervention (n = 26) (%)
Maternal age yrs (mean ± SD)	22.05 ± 5.73	24.35 ± 5.37
<18 years	2 (9 %)	1 (4 %)
18–30 years	18 (78 %)	21 (81 %)
>30 years	1 (4 %)	4 (15 %)
Parity (Primip) (%)*	21 (91 %)	6 (23 %)
Income	8 (35 %)	12 (46 %)
< $15,000	2 (9 %)	3 (12 %)
$15,000-$30,000	1 (4 %)	4 (15 %)
$30,000-$50,000	0 (0 %)	1 (4 %)
>$50,000	8 (35 %)	5 (19 %)
Do not know		
Race/Ethnicity	6 (25 %)	7 (27 %)
African American/Black	12 (52 %)	17 (65 %)
Hispanic	4 (17 %)	2 (8 %)
Other		
Marital Status: Married	3 (13 %)	7 (27 %)
Education	8 (35 %)	11 (42 %)
<12^th^ grade	6 (26 %)	5 (19 %)
High school or GED	7 (30 %)	7 (27 %)
>High school or GED		
Employment: Yes	8 (35 %)	10 (38 %)
Language spoken: English	21 (92 %)	18 (69 %)
Place of birth: United States	13 (58 %)	8 (31 %)
Public insurance	12 (52 %)	12 (46 %)
Maternal smoking (Yes, %)	5 (22 %)	6 (23 %)
Infant birth weight (Lbs) (Mean ± SD)	7.37 ± 0.83	7.36 ± 1.08
Infant sex (M, %)	12 (52)	9 (35)

Expressed as n (%) except as noted. Percentages may not add to 100 % due to rounding and missing data

*p < 0.001. All other differences were not significant

### Interventions

#### Intervention components common to NFN and NFN+ participants

Mothers who agreed to participate in the NFN program and then were eligible for ECHO and provided consent received a weekly home visit by the NFN home visitor. At the first home visit, the NFN Supervisor assessed the family’s strengths and needs using the 10-item Family Stress Scale (Kempe Scale) and tailored the NFN program using the PAT and Partners curricula (standard current practice). These curricula were then administered by home visitors. Visit documentation is kept through content logs which include information both about the visit frequency and the content of the material presented/discussed during the visit.

#### Intervention components unique to NFN+

Intervention components unique to NFN+ include: 1) enhanced support regarding breastfeeding, 2) creation of a Family Wellness Plan that teaches mothers goal-setting and self-monitoring skills to assess her family’s progress, 3) education and skill-building in behavioral strategies (problem-solving and stimulus control) to implement desired changes in four infant target areas (breastfeeding, SSB/juice consumption, introduction of solids, television/screen time and infant routines), 4) a toolkit with items useful to support the changes, and 5) linkages to community programs that support healthy behavior change.

##### Enhanced support for breastfeeding

NFN+ workers assess the mother’s attitudes and beliefs related to breastfeeding prenatally or in the hospital and provide education about the importance of breastfeeding and its benefits to the infant. After delivery, hospital staff assist new mothers in initiating breastfeeding which the NFN+ worker reinforces and supports in the home. The NFN worker observes the mother breastfeeding with special attention to the mother’s state, infant attachment, positioning, frequency, and duration of feeding. The NFN worker counsels the mother about continuing to breastfeed when returning to work and refers mothers experiencing difficulties to Heritage and Pride, a breastfeeding support program in Hartford [21, 49]. While the decision to breastfeed has often been made prior to enrollment in ECHO, barriers to breastfeeding encountered by Latinas and Blacks (e.g., comfort, lack of social support) are addressed by the NFN+ workers [50].

##### Family wellness plan

This dynamic document is created in a partnership between the mother and the NFN+ worker. NFN+ workers teach mothers SMART goal setting principles (specific, measurable, achievable, realistic, timely) and assist mothers in setting goals regarding their and their child’s health behaviors. A plan for self-monitoring behaviors, specific to the behavior (s) selected for the Family Wellness Plan, is determined. Self-monitoring calendars are used to document behaviors and are reviewed at each visit. Maternal success in achieving her goals is emphasized. The following specific behavioral targets are emphasized as goals: extending breastfeeding duration and exclusivity, no SSB/juice consumption until 6 months and then less than 6 oz/day for the next 6 months, no solids before 6 months of age and no cereal in bottles, improving parenting skills by establishing daily routines, reading infant cues related to sleep, hunger and satiety and infant physical activity (“Tummy Time”), no television viewing, and no television in the bedroom. As mothers are successful with these infant-directed targets, NFN+ workers begin to discuss ways to create a healthier food and activity environment for the mother utilizing nutrition programs (Cooking Matters^©^, visits to Farmer’s Markets/corner stores), activity programs (Yoga, Zumba) at the BFF Centers, and parenting classes. Mothers are encouraged to set small, personally meaningful and important goals and to document their goals and progress using self-monitoring calendars and a scrapbook (containing her Family Wellness Plan) created with her NFN+ worker.

##### Behavioral strategies

Modules for the core behavior skills are adapted from existing curricula (e.g. Husky Byte for SSB/juice [51, 52], Soothe/Sleep for routines [11]; Diabetes Prevention Program [53] for core behavioral skills relevant to healthy eating and activity) and include interactive activities. NFN+ workers teach mothers core behavioral skills (e.g., goal- setting) relevant to the specific behavior. Emphasis is placed on stimulus control and changing the home environment to support healthy choices (e.g., enhancing visual cues for good dietary choices).

All program materials are available in English and Spanish and have been developed and tested with home visitors, Latina and Black members of the community and parents for content, literacy and cultural relevancy. Concerns in Latina and Black mothers about weight as a sign of health [54], about being socially uncomfortable with breastfeeding [21, 55], childhood obesity as a non-issue [56], television as an educational tool [57], and outdoor safety as a barrier to exercise [58, 59] are addressed.

##### Intervention strategies

Activities within each intervention strategy module take ~10-15 min of the scheduled 60–90 min home visit and are delivered at different visits. Each targeted behavior is addressed in a series of interactive modules that can be divided into multiple 15-min segments. For example, the Sugar Sweetened Beverage Module has four “messages” linked to four activities. Message 1 stresses no juice in the first 6 months of life and comes with a bib that says “No Sugar drinks for me, I am sweet enough.” Message 2 teaches mothers to buy only 100 % fruit juice. Message 3 educates mothers about the sugar content of 100 % fruit juice and Message 4 stresses limiting juice consumption to less than 6 ounces a day from 6 months to 7 years of age. The messages build upon each other but can be delivered separately or together and in any order. NFN+ mothers are trained to recognize hunger and satiety cues in their infant and to establish sleep routines. Mothers are also taught how to use “Tummy Time” to enhance infant activity and development and how to avoid television viewing. In addition, mothers who are bottle feeding learn proper formula dilution, storage, and how to use hunger and satiety cues regardless of the milk volume consumed. The messages in each intervention module are described in Table 3.

**Table 3:** Intervention modules and messages

Intervention module	Intervention messages	Intervention activities/Components
Feeding My Baby	1. Breast milk is best	-Picture of mom breastfeeding infant
	2. Breast milk is the only food a baby needs until 6 months	-3 reasons to breastfeed
		-Importance/Confidence rulers
	3. If formula feeding, mix formula correctly	-Goal setting tool
		-Steps to achieving goal
		-Barriers/Problem solving
		-Community resources to support activity: Heritage and Pride program
		-Self-monitoring calendar
		-Progress assessment
Juice/Sugar sweetened beverages	1. No juice or SSB before 6 months of age	-Picture of baby wearing “No sugar drinks for me” bib
	2. Serve only 100% juice	-Education around messages
	3. Sugar in 100% juice	-How to read a label to determine sugar content and addition
	4. No more than 4-6 oz juice/day	-Measuring cup, 6 oz sippy cup, bib,toothbrush
		-Importance/Confidence rulers
		-Goal setting tool
		-Steps to achieving goal
		-Barriers/Problem solving
		-Community resources to support activity: Cooking Matters, Shopping Matters
		-Self-monitoring calendar
		-Progress assessment
Little Tummy	1. Delay introduction of solids to 6 months	-Picture of baby (baby’s tummy)
	2. Do not add cereal to the bottle	-Education around messages
	3. How to introduce solids	-Measuring spoons
	4. What are the first solids	-Food card “quiz”
	5. Portion sizes	-Importance/Confidence rulers
		-Goal setting tool
		-Steps to achieving goal
		-Barriers/Problem solving
		-Community resources to support activity: WIC, Fruits and vegetable coupons
		-Self-monitoring calendar
		-Progress assessment
Screen Time	1. No TV/screen time under 2 years	-Picture of mother and infant looking at book, listening to music
	2. Remove TV from bedroom	-Education around message
		-Paper and crayons to make No TV sign
		-Importance/Confidence rulers
		-Goal setting tool
		-Steps to achieving goal
		-Barriers/Problem solving
		-Community resources to support activity: Brighter Future Family Center activities
		-Self-monitoring calendar
		-Progress assessment
Establishing Routines		
A. Rock-a-bye Baby	1. Infant sleep duration	-Picture of baby sleeping
	2. Create a healthy bedtime routine	-Education around messages
		-Sleep sack
		-Importance/Confidence rulers
		-Goal setting tool
		-Steps to achieving goal
		-Barriers/Problem solving
		-Community resources to support activity: Parenting support groups
		-Self-monitoring calendar
		-Progress assessment
B. Tummy Time	1. Use tummy time to play	-Picture of baby engaging in tummy time
		-Education around benefits of tummy time for baby and mother
		-Importance/Confidence rulers
		-Playmat
		-Goal setting tool
		-Steps to achieving goal
		-Barriers/Problem solving
		-Community resources to support activity: Parenting support group
		-Self-monitoring calendar
		-Progress assessment
C. Calm Baby	1. Techniques to soothe and calm baby instead of feeding	-Picture of mother holding baby
		-Education on hunger and satiety cues; strategies to calm baby
	2. Taking care of mother when infant is crying	-Importance/Confidence rulers
		-Goal setting tool
		-Steps to achieving goal
		-Barriers/Problem solving
		-Community resources to support activity: Parenting support group
		-Self-monitoring calendar
		-Progress assessment
Healthy Mom, Healthy Baby	1. Eat 5 servings of fruits and vegetables daily	-Picture of mother
		-Education about diet and physical activity; activity with her baby
	2. Get 10,000 steps a day	-Pedometer, Fruit and Vegetable coupons
	3. Be physically active 30 minutes/day 5 days/week	-Importance/Confidence rulers
		-Goal setting tool
		-Steps to achieving goal
		-Barriers/Problem solving
		-Community resources to support activity: Exercise classes, Cooking Matters, Shopping Matters
		-Self-monitoring calendar
		-Progress assessment

All modules have a similar format. Modules begin with introduction of a topic and the recommendation followed by education related to the rationale for the recommendation. The importance to the mother and her confidence to implement the recommendations are then explored using importance and confidence rulers. This is followed by the mother creating a goal for herself or her infant related to the module and problem-solving around the goal. A list of community linkages and a self-monitoring calendar are then provided by the NFN+ worker. The calendar and use of these community linkages are reviewed with the mother in later visits and the goals are reinforced. If a mother has been unsuccessful in achieving her goal, the goal is reviewed and revised with new problem-solving strategies and a new self-monitoring calendar is completed for subsequent review.

##### Toolkit

Mothers are provided with different items related to each module and a picture of their infant engaged in the activity is taken to encourage use. For example, for the juice modules, a measuring cup and 6 oz sippy cup are provided in addition to the bib described previously. For Tummy Time, a play mat is provided while no television is encouraged through creation of a sign that is placed on the television.

##### Linkages to relevant community programs

Each intervention module includes linkages to relevant community programs (e.g., Heritage and Pride for breastfeeding; BFF Centers for Yoga/Zumba and parenting classes, Cooking Matters^©^, Shopping Matters^©^, Husky Byte, FoodShare, Hartford Food System, and to WIC-approved and ECHO participating grocery/corner stores that accept program-created coupons for fruits and vegetables). When needed or appropriate, childcare and bus tokens are provided to facilitate use of these community programs. Intervention mothers are provided with fruit and vegetable coupons ($3/week) that they can use at local grocery stores and Farmers Markets. Mothers are provided with a pedometer to help track their steps each day. In addition, mothers can choose a “bonus” item such as cookware, a water filter system, bus passes, running shoes for themselves or a stroller. These bonus items were chosen because NFN workers noted that families were not able to participate in various activities such as cooking or exercise classes or walking activities because they didn’t have necessary items.

### Training

#### Training common to NFN and NFN+

All NFN and NFN+ Supervisors received training in the ethical conduct of human research and are certified. Additionally, they received two hours of training in how to obtain consent for the ECHO study and in how to complete each of the program’s questionnaires. Study staff accompanied the supervisors during the initial home visit to support the supervisors in obtaining consent and in completing the questionnaires.

#### Training unique to NFN+

NFN+ Supervisors and home visitors received 5 h of initial training and twice yearly formal booster sessions in ECHO including how to engage mothers in the behavioral change strategies and in how to render each of the intervention modules. They received an additional 4 h of training on the importance of breastfeeding, common breastfeeding problems, tips for successful breastfeeding and how to recognize abnormal breastfeeding by the Heritage and Pride program staff. A list of available resources to support mothers with breastfeeding and how to access these resources was provided. A study coordinator makes weekly visits to intervention centers to review the intervention module prior to delivery and to debrief with NFN staff after a home visit to assure consistency and completeness of the rendered intervention.

### Outcome measures

#### Demographics

Basic demographic information is obtained by self-report from mothers and is included in Table 1.

#### Process or intermediary variables

Message-specific maternal knowledge, intention and self-efficacy is being assessed using the Infant Feeding Practice Survey II [60, 61] adapted for prenatal and postnatal feeding practices and the Infant Feeding Style Questionnaire [62].

#### Primary outcome variables

Breastfeeding extent and duration and feeding behaviors related to solids and juices and sugar sweetened beverages are being assessed using questions from the Infant Feeding Practices Survey II and the Infant Feeding Style Questionnaire. Questions related to soothability have been extracted with permission from the Infant Behavior Questionnaire-Revised [63] and are administered at birth, 6 and 12 months. The Brief Infant Sleep Questionnaire [64] is being used to assess sleep patterns and duration. Questions about infant activity and television viewing are being used from other published studies [65, 66].

#### Secondary outcome variables

Maternal diet and behavior are being assessed using self- report on maternal fruits, vegetables, and beverage consumption [67]. Mothers are asked how many days per week they eat breakfast, lunch, dinner, and snacks and how many days per week they eat at fast-food or other restaurants. Information about the food and beverages in the home is obtained. The Paffenbarger Health Fitness Assessment is being used to assess maternal activity [68]. Infant weight and length with calculation of the weight for length *z*-score are determined by medical record review at birth, 6 and 12 months.

#### Additional variables

Three potential moderators of the treatment response are being assessed including the 4-item Perceived Stress Scale [69], the Family Social Support Questionnaire [70], and the Edinburgh Maternal Depression Screening Tool [71].

#### Additional measures in intervention arm

Doses of the intervention are being assessed using the home visitor logs and documented debriefings of home visits by home visitors with study staff, the number of returned fruits and vegetables coupons, documentation of activity in the Family Wellness Plan workbook and sign-in logs for activities at the Brighter Future Family Centers. Maternal satisfaction with the intervention and with specific modules in the intervention is being assessed using a satisfaction survey delivered by telephone by study staff at 6 and 12 months.

### Evaluation plan

**Fig. 3:**
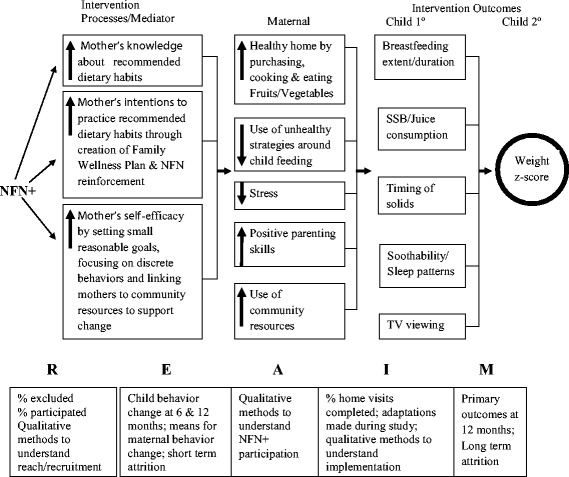
Analytic plan for ECHO study

Assessments will occur at birth and when the infant is 6 and 12 months. The Family Wellness Plan book is collected at 6 and 12 months and copied and returned to the family. Primary outcomes will be the child’s targeted behaviors at 12 months (i.e., extent and duration of breastfeeding, SSB consumption, timing of introduction of solids, sleep duration and soothability and television viewing) (Fig. 3). Assessed process variables include changes in mothers’ knowledge about dietary recommendations, intentions to make these changes, and self-efficacy to make healthy choices. Six month intermediary variables include changes in the home food environment and mothers’ shopping and cooking practices, child feeding styles, maternal stress, positive parenting practices, and community resource utilization. The RE-AIM analytic framework will be used [72, 73]. For RE-AIM, Reach will be explored by determining the percent of mothers/infants who were excluded and the reasons for exclusion, the percent of eligible mothers/infants who participated, and reasons for dropping out. Effectiveness will be evaluated by comparing differences in maternal behaviors at 6 months between control and intervention mothers and short-term attrition. For Adoption, intervention use (number of doses of each intervention and number of intervention types rendered) and NFN+ worker satisfaction and intention to use the intervention after study’s end will be evaluated. Implementation will be evaluated using the number of completed home visits and the association between these indicators and maternal behaviors. Maintenance will be assessed by examining child behaviors and weight for length *z*-scores at 12 months.

Threats to internal validity include family mobility, the population and neighborhood comparability, and their representativeness. To address these threats, we have limited eligibility criteria, paired the neighborhoods using important variables, and have sequentially enrolled eligible mothers to the target number per neighborhood. Hartford families return to the same Brighter Future Family Center and to the same clinic (e.g., <1 % change clinics each year [74]) and the NFN home visitor will provide continuity if the family moves but remains in the local area. Other threats include repeated testing and NFN+ worker bias. Where possible, we will use both objective (e.g., coupon use) and subjective (e.g., self-reported fruit and vegetable consumption) measures and will compare NFN and NFN+ home visit logs for content and visit frequency. Additional threats include NFN staff turnover. Threats to external validity include the patient population, attrition in the NFN program and the urban community.

#### Sample size, power calculation

This is an initial efficacy trial that is constrained by the size of the budget. However, 60 participants with a 20 % drop out rate is sufficient to detect a decrease of approximately 0.3 in the proportion of children under 6 months of age receiving SSB/juice and/or solid food with a power of 80 % and an alpha = 0.05.

### Statistical analysis plan for primary outcomes

The primary outcome measure is the duration and exclusivity of breastfeeding, the time to consumption of SSB/juice and solids, the amount of television/screen time per day, and sleep duration in NFN+ infants as compared to NFN infants. The quality of the randomization and the comparability of the participants have been assessed using >Chi square and t-tests as appropriate (Table 2). Participants were comparable except that intervention participants had higher parity as compared to control participants. Participants are nested and clustered within neighborhoods and these effects are being evaluated and will be controlled for in the statistical analysis. Using an intention to treat and a per protocol analysis, behaviors of NFN+ mothers will be compared to NFN mothers related to SSB/juice consumption (number of ounces per day, age at introduction of SSB/juice; age at beginning solids, duration (weeks) of breastfeeding and sleep duration, soothability, and television viewing hours) using two sample t-tests or Wilcoxon tests. Chi square analysis will be used to compare the proportions of exclusive breastfeeding at 6 months in the two groups. Similarly, we will determine if the intervention decreases the risk that infants would stop exclusive breastfeeding at any time considered in the study. A p ≤ 0.05 will be considered significant.

#### Statistical analysis plan for secondary outcomes

Secondary aims which compare NFN+ to NFN mothers related to a) consumption of fruits and vegetables and b) utilization of the Brighter Future Family Centers will be analyzed similar to the primary hypotheses. For the comparison of weight for length *z-*score between NFN+ and NFN infants, weight categories based on weight for length percentiles will be compared using chi-square analysis.

## Discussion

New models and interventions to prevent childhood obesity and reverse the staggering increases in obesity prevalence over the past 40 years are needed. Interventions need to not only be successful but to also be capable of wide dissemination and implementation at reasonable cost.

The Early Childhood Obesity Prevention Program (ECHO) is a randomized controlled trial testing the efficacy of the early establishment of healthy behaviors in preventing childhood obesity in mothers of Latino and Black children from a low-income, urban community. It is an innovative, ecologically- and culturally-based intervention to obesity prevention. The intervention builds upon an existing home visitation program by incorporating maternal education with skill-building (goa-setting, problem-solving, self-monitoring, and stimulus control). The intervention then uses these core behavioral strategies to target 4 specific areas of behavior change (breastfeeding, delayed introduction of solids, delayed consumption of fruit juice, and infant routines including limiting television/screen time, recognizing cues for hunger and satiety and sleep duration) that mothers are open to change. The intervention has been designed to be incorporated into ongoing home visits which will enhance opportunities for wide dissemination. The intervention is also grounded in Behavior Theory and the Ecological Model of Childhood Obesity to activate mothers to implement new behaviors in the home. The intervention links mothers to community programs and resources, supports maternal changes, and provides feedback on achievement of specific goals determined by the mother within a culturally responsive framework. It also addresses concerns in Latina and Black mothers about weight as a sign of health, breastfeeding, television as an educational tool, and outdoor safety. The study is innovative in its focus on newborns, the use of mothers as agents of change, and the utilization of an existing home visitation program to enhance mother-infant outcomes. The intervention also sets the stage for lasting and endurable change by taking into account the child’s environmental and social milieu and connecting families to neighborhood programs and resources to support behavior change.

## Abbreviations

NFN: Nurturing Families Network
NFN+: Enhanced NFN curriculum
PAT: *Parents as Teachers-Born to Learn*
Partners: *Partners for a Healthy Baby*
SSB: Sugar-sweetened beverages
BFF Center: Brighter Future Family Center
